# NASHmap: clinical utility of a machine learning model to identify patients at risk of NASH in real-world settings

**DOI:** 10.1038/s41598-023-32551-2

**Published:** 2023-04-05

**Authors:** Jörn M. Schattenberg, Maria-Magdalena Balp, Brenda Reinhart, Andreas Tietz, Stephane A. Regnier, Gorana Capkun, Qin Ye, Jürgen Loeffler, Marcos C. Pedrosa, Matt Docherty

**Affiliations:** 1 Metabolic Liver Research Program, I. Department of Medicine, University Medical Center, Mainz, Germany; 2 Novartis Pharma AG, Basel, Switzerland; 3 ZS Associates, Zurich, Switzerland; 4 ZS Associates, Philadelphia, PA USA

**Keywords:** Gastroenterology, Medical research, Health services, Public health, Computational science, Information technology, Scientific data

## Abstract

The NASHmap model is a non-invasive tool using 14 variables (features) collected in standard clinical practice to classify patients as probable nonalcoholic steatohepatitis (NASH) or non-NASH, and here we have explored its performance and prediction accuracy. The National Institute of Diabetes and Digestive Kidney Diseases (NIDDK) NAFLD Adult Database and the Optum Electronic Health Record (EHR) were used for patient data. Model performance metrics were calculated from correct and incorrect classifications for 281 NIDDK (biopsy-confirmed NASH and non-NASH, with and without stratification by type 2 diabetes status) and 1,016 Optum (biopsy-confirmed NASH) patients. NASHmap sensitivity in NIDDK is 81%, with a slightly higher sensitivity in T2DM patients (86%) than non-T2DM patients (77%). NIDDK patients misclassified by NASHmap had mean feature values distinct from correctly predicted patients, particularly for aspartate transaminase (AST; 75.88 U/L true positive vs 34.94 U/L false negative), and alanine transaminase (ALT; 104.09 U/L vs 47.99 U/L). Sensitivity was slightly lower in Optum at 72%. In an undiagnosed Optum cohort at risk for NASH (n = 2.9 M), NASHmap predicted 31% of patients as NASH. This predicted NASH group had AST and ALT mean levels above normal range of 0–35 U/L, and 87% had HbA1C levels > 5.7%. Overall, NASHmap demonstrates good sensitivity in predicting NASH status in both datasets, and NASH patients misclassified as non-NASH by NASHmap have clinical profiles closer to non-NASH patients.

## Introduction

Nonalcoholic fatty liver disease (NAFLD) is the most common chronic liver disease, with a worldwide prevalence of approximately 20–35% depending on the study population and diagnostic criteria used^1^. Key risk factors associated with NAFLD are obesity, diabetes, hypertension, and metabolic syndrome^2^, all of which are on the rise worldwide, leading to ever increasing new cases of NAFLD. Excess lipid accumulation within hepatocytes in NAFLD patients can lead to an increase in inflammatory processes, causing disease progression to nonalcoholic steatohepatitis (NASH). Over time, liver inflammation in NASH patients can lead to hepatocyte oxidative damage (e.g., lipid peroxidation) and cell death, liver fibrosis, and end-stage liver disease (cirrhosis and hepatocellular carcinoma [HCC])^3,4^. NASH is associated with significantly lower health-related quality of life than NAFL^5^ and high cost-of-illness^6^. However, NASH (with or without fibrosis) remains largely under-diagnosed because of the lack of specific clinical symptoms, low awareness among patients and lack of treatments approved specifically for NASH. Identifying patients with a high probability of NASH is the first step towards risk stratification and diagnosis for disease management.

A confirmed diagnosis of NASH depends on an invasive liver biopsy with inherent risks to the patient. Therefore, biopsies are typically reserved for symptomatic patients with advanced disease^4,7^. A non-invasive XGBoost (eXtreme Gradient Boosting) model^8^ called NASHmap has been developed to classify patients as NASH or non-NASH using 14 variables commonly collected in standard clinical practice. In testing, 81% of NASH patients were correctly identified in a general population and 86% in a type 2 diabetes mellitus (T2DM) subcohort^8^. NASHmap provides a reliable, non-invasive, easy-to-use tool to supplement healthcare provider decisions to screen patients for probable NASH. For providers and researchers to leverage NASHmap more effectively, it is essential to understand the characteristics of patients correctly and incorrectly classified by the model in different populations.

Here, we profiled patients based on their NASHmap classification and explored potential causes of NASHmap classification errors. In addition, we examined the utility of NASHmap in a real-world setting by comparing profiles of patients with clinician-diagnosed NASH to undiagnosed patients predicted to have NASH by NASHmap in both general and at-risk populations.

## Methods

### Data sources

Data were obtained from the US National Institutes of Diabetes and Digestive Kidney Diseases NAFLD Adult Database (NIDDK; 2004–2009) and the Optum de-identified Electronic Health Record Database (Optum; 2007–2017). The NIDDK NAFLD Adult Database enrolled adult US patients with the full spectrum of NAFLD or cryptogenic cirrhosis and other causes of liver disease excluded. Data including demographic information (e.g., age, BMI), histological results (e.g., steatosis, ballooning) and clinical laboratory test results were collected longitudinally over a 4-year period from 2002 to 2006 with a median follow up of 2.1 years. The Optum database contains approximately 86 million electronic health records (EHR) obtained from 150,000 healthcare providers, 2,000 hospitals, and 7,000 clinics in the United States, collected over a 10-year period from January 2007 through December 2017. Standard clinical information collected during routine patient visits includes patient demographics, diagnoses, procedures, laboratory results, and prescription medication data.

### Cohort selection and NASHmap classification

#### NIDDK NAFLD adult cohort

The cohort with biopsy-confirmed NAFLD or NASH and no other diagnosed liver diseases was initially split in a training and a test cohort, with the training cohort used for the development of NASHmap^8^. Only the test cohort was used for the current study (see Supplemental Materials for additional details). The index date was the date of the most recent liver biopsy used to determine NASH status. Data for NASHmap predictions were selected from the baseline visit report at study enrollment (presence of hypertension, weight, height) and the closest visit within ± 6 months of the index date (laboratory test values). Missing values for features of the model were imputed from other values in the dataset (see “Statistical methods” below). The following subcohorts were created and used for analysis: (1) biopsy-confirmed NASH and non-NASH as determined by the most recent liver biopsy results or (2) T2DM present and no-T2DM as reported during the baseline visit.

#### Optum cohort

Patients ≥ 18 years of age with NASH, NAFLD, and/or NAFLD associated conditions and with data for all 14 features of NASHmap available in a 6-month window (index window) were selected (see Supplemental Materials for details). Patients with any other liver conditions were excluded. NAFLD associated conditions were defined as a diagnosis (cirrhosis, fibrosis, hepatocellular carcinoma or a comorbid condition such as T2DM, hypertension, hyperlipidemia, polycystic ovary syndrome) or a procedure indicating liver disease (liver biopsy, liver transplant, or bariatric surgery). Presence and absence of disease diagnoses or procedures were based on International Classification of Diseases 9th or revision (ICD-9 or ICD-10), and Current Procedural Terminology (CPT) respectively. Data from the most recent index window was used for NASHmap predictions, and the mean value for a feature was used if multiple values existed in the window.

Subcohorts for analysis were created based on (1) NASH status: biopsy-confirmed NASH (patients with an ICD-10 K75.81 NASH code and liver biopsy); ICD-10 NASH (patients with an ICD-10 K75.81 NASH code excluding the biopsy-confirmed NASH); and undiagnosed (patients with no record of NASH or NAFLD ICD-9 code 571.8 and ICD-10 codes K76.0 and K75.81) or (2) T2DM status: presence or absence (by ICD-9 or ICD-10 code).

### Statistical methods

#### Model classification errors and associated patient characteristics

The NASHmap model^8^ uses 14 clinical and laboratory variables (named features hereafter): HbA1c; AST (units/L); ALT (units/L); total protein (g/dl); AST/ALT; BMI (kg/m^2^); triglycerides (mg/dl); height (cm); platelets (cell/μl); white blood cells (1000 cells/μl); hematocrit (%); albumin (g/dl); hypertension (Y/N); and gender. NASHmap was applied in the NIDDK NAFLD study test cohort (stratified by NASH and T2DM status respectively) and confusion matrices were generated showing the 4 possible patient classification groups: *true positives* (clinical confirmed NASH, predicted NASH), *false positives* (clinical non-NASH, predicted NASH), *true negatives* (clinical non-NASH and predicted non-NASH), and *false negatives* (clinical NASH, predicted non-NASH). Sensitivity, or true positive rate, was calculated as the proportion of NASH patients correctly predicted out of the total number of confirmed NASH patients.

Data for all 14 features is required for NASHmap. To assess the impact of imputation of missing data on NASHmap performance, actual values for HbA1c, the feature with highest predictive power, were removed for all patients in the NIDDK test cohort and replaced with imputed values using three methods: K-nearest neighbor (kNN), median value for HbA1c in the cohort, and mean value for HbA1c in the cohort. kNN replaces missing values with the mean value of the feature from the k most similar neighbors for data imputation, and k = 5 was used^8^. If data are only missing for a subset of patients, patient level imputation through kNN or similar methods could reduce the impact of missing data. If all values are missing, imputation with a reasonable fixed value such as a population median or mean is possible but will impact model performance. NASHmap performance for each imputation method was compared using the area under the curve (AUC) metric.

Summary statistics were used to compare NASHmap feature values between clinical subcohorts and NASHmap classification groups to explore potential causes of prediction errors. For the Optum study cohort, comparisons were made between patients in clinically defined categories and NASHmap predicted categories. Patient clinical and laboratory data were expressed as mean ± SD. Differences in group means were assessed by either T-tests for continuous variables and chi-square tests for categorical variables or, for groups with a large imbalance of patient numbers, by percentage of values outside normal range (see Supplemental Table 1 for definitions). Analyses were performed using R.

## Results

### Performance of NASHmap in NIDDK patients with biopsy-confirmed NASH status

The test cohort used to test the performance of NASHmap in the NIDDK NAFLD Adult Database comprised 281 patients with 181 patients having biopsy-confirmed NASH and 100 biopsy-confirmed non-NASH (Table 1).

**Table 1:** Clinical characteristics of study cohorts by clinical status.

Characteristics	NIDDK		Optum		
	NASH (n = 181)	Non-NASH (n = 100)	NASH (n = 1016)	ICD-10 NASH (n = 21,930)	Undiagnosed NASH (n = 2,886,653)
Age, mean ± SD	48.6 ± 10.4	48.2 ± 9.4	55.5 ± 12.6	57.3 ± 13.2	61.2 ± 15.3
Female, N (%)	124 (69%)	52 (52%)	663 (65%)	13,168 (60%)	1,544,078 (53%)
T2DM, N (%)	84 (46%)	28 (28%)	721 (71%)	14,916 (68%)	1,532,744 (53%)
Hypertension, N (%)	101 (56%)	44 (44%)	805 (79%)	16,948 (77%)	2,253,961 (78%)
Hyperlipidemia, N (%)	110 (61%)	53 (53%)	803 (79%)	17,558 (80%)	2,101,467 (73%)
BMI, mean ± SD	33.8 ± 5.2	33.4 ± 6.0	34.8 ± 7.3	35.5 ± 8.1	32.7 ± 45.2
HbA1c, mean ± SD	6.6 ± 1.8	5.8 ± 1.0	6.7 ± 1.6	6.7 ± 1.6	6.6 ± 16.0

NIDDK: National Institute of Diabetes and Digestive Kidney Diseases; NASH: biopsy-confirmed NASH; Non-NASH: biopsy-confirmed non-NASH; ICD-10 NASH: patient coded for NASH, no biopsy reading; undiagnosed NASH: patients with no records of NASH by coding or liver biopsy; T2DM: type 2 diabetes mellitus; BMI: body mass index; HbA1c: glycated hemoglobin.

NASHmap performance as assessed by area under the curve (AUC) was 0.82, accuracy was 75% (210/281), sensitivity was 81% (147/181), precision or positive predictive value (PPV) was 80% (147/184), and negative predicted value (NPV) was 65% (63/97)^8^. When the key feature HbA1c was considered missing and imputed for the test patients (N = 281), AUC was 0.79 using K-nearest neighbor (kNN) imputation, 0.77 using the median dataset value, and 0.76 using the mean dataset value.

NASHmap showed a good performance in correctly classifying patients according to their clinical status: 81% (147/181) of NASH patients and 63% (63/100) of non-NASH patients were correctly classified as NASH and non-NASH respectively (Table 2). Good performance was also achieved in the cohort stratified by T2DM status. NASH was correctly predicted in 86% (72/84) of NASH patients with T2DM as compared to 77% (75/97) of NASH patients without T2DM (Table 3).

**Table 2:** Performance of NASHmap in classifying patients in a cohort with known status from NIDDK.

		Predicted class (NASHmap)		Metrics
		Positive	Negative	
True class (biopsy confirmed NASH)	Positive	TP = 147	FN = 34	Sensitivity/TPR = 81%
	Negative	FP = 37	TN = 63	Specificity/TNR = 63%
Metrics		Precision/PPV = 80%	NPV = 65%	Accuracy = 75%

Accuracy: TP + TN/(TP + FP + FN + TN); FN: false negative; FP: false positive; NIDDK: National Institute of Diabetes and Digestive Kidney Diseases; NASH: nonalcoholic steatohepatitis; NPV: negative predictive value (TN/[FN + TN]); PPV: positive predictive value (TP/[TP + FP]); TN: true negative; TNR: true negative rate (TN/[FP + TN]); TP: true positive; TPR: true positive rate (TP/[TP + FN]).

**Table 3:** Performance of NASHmap in classifying patients in the NIDDK cohort stratified by T2DM status.

			Predicted class (NASHmap)		Metrics
			Positive	Negative	
T2DM (N = 112)	True class (biopsy confirmed NASH)	Positive	TP = 72	FN = 12	Sensitivity/TPR = 86%
		Negative	FP = 10	TN = 18	Specificity/TNR = 64%
	Metrics		Precision/PPV = 88%	NPV = 60%	Accuracy = 80%
Non-T2DM (N = 169)	True class (biopsy confirmed NASH)	Positive	TP = 75	FN = 22	Sensitivity/TPR = 77%
		Negative	FP = 27	TN = 45	Specificity/TNR = 63%
	Metrics		Precision/PPV = 74%	NPV = 67%	Accuracy = 71%

Accuracy: TP + TN/(TP + FP + FN + TN); FN: false negative; FP: false positive; NIDDK = National Institute of Diabetes and Digestive Kidney Diseases; NASH = nonalcoholic steatohepatitis; NPV: negative predictive value (TN/[FN + TN]); PPV: positive predictive value (TP/[TP + FP]); TN: true negative; TNR: true negative rate (TN/[FP + TN]); TP: true positive; TPR: true positive rate (TP/[TP + FN]).

To explore potential causes of NASHmap errors, we compared mean values of each of the 14 features in each classification group. In the biopsy-confirmed NASH patient subcohort, the patients correctly classified (true positive) by NASHmap had clinical profiles consistent with NASH (Table 4). For example, mean ± SD values for AST and ALT in this group were 75.88 ± 49.64 U/L and 104.09 ± 52.77 U/L, respectively, far outside of the normal upper limits of 35 U/L. Incorrectly classified (false negative) patients had clinical profiles closer to non-NASH for several features, with significantly lower mean values of HbA1C, AST, ALT, total protein and albumin than true positive NASH (Table 4 and Supplemental Fig. 1). Table 4 shows the clinical variables in the order of their predictive importance in NASHmap. Of the five features with highest predictive value (HbA1C, AST, ALT, total protein and AST/ALT ratio), three (AST, ALT, and total protein) had statistically significant differences between true positive and false negative patients (Table 4).

**Table 4:** Features of patients correctly classified and misclassified by NASHmap in NIDDK.

NASHmap feature^a^	Biopsy-confirmed NASH			Biopsy-confirmed non-NASH		
	True positive (n = 147)	False negative (n = 34)	*P*-value	True negative (n = 63)	False positive (n = 37)	*P*-value
HbA1c (%)	6.72 ± 2.09	6.04 ± 1.64	0.04	5.53 ± 0.85	6.13 ± 1.23	0.01
AST (U/L)	75.88 ± 49.64	34.94 ± 16.20	< 0.01	38.12 ± 20.11	53.76 ± 21.43	< 0.01
ALT (U/L)	104.09 ± 52.77	47.99 ± 44.08	< 0.01	44.77 ± 20.62	82.57 ± 55.55	< 0.01
Total protein (g/dL)	7.46 ± 0.61	7.15 ± 0.47	< 0.01	7.07 ± 0.48	7.36 ± 0.68	0.03
AST/ALT	0.82 ± 0.30	0.94 ± 0.48	0.16	0.94 ± 0.45	0.77 ± 0.30	0.02
BMI	33.42 ± 4.68	35.49 ± 6.88	0.87	33.51 ± 6.22	33.25 ± 5.60	0.17
TG (mg/dL)	219.09 ± 147.20	205.53 ± 144.93	0.63	148.85 ± 75.00	156.87 ± 103.04	0.68
Height (cm)	167.00 ± 8.17	167.83 ± 9.57	0.87	167.83 ± 7.21	166.72 ± 9.71	1.00
PLT (× 10^3^/μL)	237.63 ± 77.39	225.08 ± 73.34	0.36	247.09 ± 72.05	251.08 ± 67.56	0.78
WBC (× 10^3^/μL)	7.03 ± 2.04	7.25 ± 2.12	0.58	6.55 ± 1.59	6.83 ± 2.12	0.47
Hematocrit (%)	41.99 ± 4.24	41.87 ± 3.60	0.87	41.86 ± 3.52	41.44 ± 3.39	0.56
Albumin (g/dL)	4.37 ± 0.36	4.09 ± 0.34	< 0.01	4.19 ± 0.41	4.33 ± 0.34	0.07
Hypertension, n (%)	81 (55.10%)	20 (58.82%)	0.84	30 (47.62%)	14 (37.84%)	0.46
Female, n (%)	105 (71.43%)	21 (61.76%)	NA	32 (50.79%)	19 (51.35%)	NA

NIDDK: National Institute of Diabetes and Digestive Kidney Diseases; NASH: nonalcoholic steatohepatitis; HbA1c: glycated hemoglobin; AST: aspartate transaminase, ALT: alanine transaminase; BMI: body mass index; TG: triglycerides; PLT: platelets; WBC: white blood cells.

^a^Features are listed by decreasing order of predictive importance in the model. Data are presented as mean ± SD unless otherwise stated.

Similarly, the group of biopsy-confirmed non-NASH patients incorrectly classified as NASH (false positive) had clinical profiles consistent with their NASHmap prediction, with mean values for HbA1C, AST, ALT, and total protein significantly increased as compared to correctly classified patients (true negatives). All five features with the highest predictive value had statistically significant differences between true negatives and false positives (Table 4).

Metabolic comorbidities such as hypertension and obesity which are frequently present in NASH^1,9,10^ do not appear to correlate with misclassification of patients by NASHmap. BMI, triglyceride levels, and rate of hypertension were all similar between true positives and false negatives. Interestingly, rates of hypertension were the lowest in the false positive group (Table 4).

### Performance in real-world Optum electronic health records

In the Optum database, 13.72 million patients met the inclusion and exclusion criteria and 3.14 million patients had data for all 14 components of NASHmap available. Among them, 1,016 patients had biopsy-confirmed NASH, 21,930 patients had a NASH ICD-10 diagnosis with no record of liver biopsy and 2,886,653 were undiagnosed patients (Table 1). Patients with liver-biopsy confirmed NASH and ICD-10 NASH showed a comparable clinical profile while the undiagnosed patients were on average older with a slightly lower BMI, a lower percentage of T2DM and lower mean HbA1C as compared to the NASH cohorts. The non-NASH and undiagnosed patient subcohort from NIDDK and Optum appeared to have lower average BMI and fewer comorbid conditions associated with NASH (Table 1).

Among Optum patients with biopsy-confirmed NASH, 72% (727/1016) were correctly classified by NASHmap (Table 5), a lower performance than in the NIDDK test cohort where 81% (147/181) of NASH patients were correctly classified (Table 2). AUC was also slightly lower at 0.76^8^. Irregular feature capture and reliance on medical coding for diagnoses in real-world databases may account for some of this difference. Reasons for NASHmap misclassification of Optum patients are consistent with those for NIDDK patients; the group of false negative patients have clinical profiles with values more likely to be within normal ranges as compared to true positives. For example, the false negative group had mean ± SD values for AST of 27.63 ± 19.85 U/L and for ALT of 30.55 ± 22.93 U/L, while the true positive group values were 66.63 ± 81.18 U/L and 72.22 ± 59.19 U/L, respectively. A substantially greater proportion of true positives had feature values outside of the normal range as compared to false negatives (% difference, true positive % − false negative %), with the greatest differences for HbA1C (32%), AST (53%), and ALT (51%) (Table 5).

**Table 5:** Clinical features of Optum real-world database patients predicted as NASH or non-NASH by NASHmap.

NASHmap feature	Biopsy-confirmed NASH				Undiagnosed			
	True positive (n = 727)	%ONR	False negative (n = 289)	%ONR	Predicted NASH (n = 883,867)	%ONR	Predicted non-NASH (n = 2,002,786)	%ONR
HbA1c (%)	6.98 ± 1.62	81	6.03 ± 1.45	49	7.06 ± 1.72	87	6.31 ± 1.53	63
AST (U/L)	63.63 ± 81.18	69	27.63 ± 19.85	16	41.21 ± 144.28	28	21.27 ± 35.24	3
ALT (U/L)	72.22 ± 59.19	76	30.55 ± 22.93	25	42.44 ± 99.86	41	23.35 ± 28.51	11
Total protein (g/dL)	7.35 ± 0.6	17	6.94 ± 0.63	8	7.28 ± 3.23	13	6.95 ± 1.42	6
AST/ALT	0.96 ± 0.46	32	1.00 ± 0.48	39	1.08 ± 0.64	43	1.03 ± 0.65	41
BMI	35.36 ± 7.14	ND	33.26 ± 7.62	ND	34.43 ± 8.18	ND	31.80 ± 7.91	ND
TG (mg/dL)	200.99 ± 171.42	23	152.35 ± 263.85	8	197.16 ± 177.68	22	131.53 ± 100.02	6
Height (cm)	167.46 ± 9.67	NA	168.07 ± 10.24	NA	168.56 ± 10.62	NA	168.92 ± 10.71	NA
PLT (× 10^3^/μL)	213.76 ± 81.6	21	221.44 ± 87.32	20	240.08 ± 76.36	8	244.12 ± 73.59	7
WBC (× 10^3^/μL)	7.42 ± 2.5	14	6.75 ± 2.49	10	8.47 ± 3.92	22	7.51 ± 3.32	13
Hematocrit (%)	40.37 ± 4.96	13	39.01 ± 5.87	16	40.77 ± 5.42	12	39.89 ± 5.19	12
Albumin (g/dL)	4.01 ± 0.54	14	3.88 ± 0.62	23	4.05 ± 4.33	11	3.92 ± 5.08	17
Hypertension, n (%)	590 (81.16%)	NA	215 (74.39%)	NA	723,571 (81.86%)	NA	1,530,390 (76.41%)	NA
Female, n (%)	476 (65.47%)	NA	187 (64.71%)	NA	460,981 (52.16%)	NA	1,083,097 (54.08%)	NA

Data are presented as mean ± SD unless otherwise stated.

NASH: nonalcoholic steatohepatitis; %ONR: % out of normal range; ND: not determined; NA: not applicable; HbA1c: glycated hemoglobin; AST: aspartate transaminase, ALT: alanine transaminase; BMI: body mass index; TG: triglycerides; ONR: outside normal range; PLT: platelets; WBC: white blood cells.

### NASHmap prediction of NASH among undiagnosed NASH patients in the Optum database

All undiagnosed patients in the Optum subcohort selected for this study have potential for NASH due to presence of comorbid conditions, yet none had an ICD code for NASH or NAFLD diagnosis in their electronic medical records (see “Methods” and Supplemental Methods). Therefore, NASHmap was used to determine the number of predicted NASH patients among these undiagnosed patients. Approximately 31% (883,867 out of 2,886,653) were predicted to have NASH (Table 5). Unlike the predicted non-NASH patients, these predicted NASH patients have mean AST and ALT levels above normal range (41.21 ± 144.28 U/L and 42.44 ± 99.86 U/L), and 66% (585,272/883,867) had T2DM in contrast to 53% (1,532,744/2,886,653) in the overall undiagnosed population.

In the subcohort of undiagnosed patients with T2DM, 38% (585,272 out of 1,532,744) were predicted as NASH, which is a slightly higher percentage than in the overall population of undiagnosed patients (Table 6). Among the T2DM cohort, there were few clinical differences between predicted NASH and biopsy-confirmed NASH patients. Among NASHmap predicted patients, 51% were female as compared to 66% of biopsy-confirmed NASH patients. Fewer values outside of normal range were found for AST and ALT in NASH predicted patients as compared to biopsy-confirmed NASH patients (% difference, true positive % − false negative %): AST (27%) and ALT (21%). However, AST/ALT ratio was out of normal range in a slightly higher number of predicted NASH patients (43%) than in biopsy-confirmed NASH patients (37%).

**Table 6:** Profile of patients with biopsy-confirmed NASH and predicted NASH in an Optum database T2DM cohort.

NASHmap feature	T2DM + confirmed NASH (n = 721)	%ONR	T2DM + predicted NASH (n = 585,272)	%ONR
HbA1c (%)	7.17 ± 1.71	84	7.66 ± 1.77	96
AST (U/L)	53.03 ± 78.22	53	40.09 ± 175.92	26
ALT (U/L)	57.24 ± 51.33	60	40.52 ± 96.02	39
Total protein (g/dL)	7.21 ± 0.63	13	7.26 ± 7.48	12
AST/ALT	0.99 ± 0.46	37	1.07 ± 0.64	43
BMI	35.09 ± 7.40	74	34.75 ± 28.50	71
TG (mg/dL)	196.93 ± 232.16	19	208.48 ± 697.51	24
Height (cm)	167.25 ± 9.83	NA	168.78 ± 10.66	NA
PLT (× 10^3^/μL)	208.29 ± 86.68	25	238.66 ± 147.18	8
WBC (× 10^3^/μL)	7.26 ± 2.54	13	8.57 ± 7.48	23
Hematocrit (%)	39.49 ± 5.30	16	40.63 ± 26.38	12
Albumin (g/dL)	3.93 ± 0.58	18	4.02 ± 5.13	11
Hypertension, n (%)	623 (86.41%)	NA	502,466 (85.85%)	NA
Female, n (%)	476 (66%)	NA	296,032 (51%)	NA

Data are presented as mean ± SD unless otherwise stated.

NASH: nonalcoholic steatohepatitis; HbA1c: glycated hemoglobin; AST: aspartate transaminase, ALT: alanine transaminase; BMI: body mass index; NA: not applicable; ONR: outside normal range; PLT: platelets; T2DM: type 2 diabetes mellitus; TG: triglycerides; WBC: white blood cells.

## Discussion

NASHmap was developed and validated in cohorts from the NIDDK and Optum EHR databases, and the model showed good performance in predicting probable NASH^8^. In this study, we further explored the prediction performance of NASHmap and compared profiles of various subcohorts of patients to understand the model predictions. Implementation of NASHmap in a real-world electronic heath record database revealed that many patients may have NASH but have no coded diagnosis. Even high-risk patients, such as T2DM patients with abnormal laboratory and clinical parameters, may not be suspected of NASH and referred for proper evaluation, illustrating the urgent need for a simple tool based on common clinical characteristics and laboratory tests to predict NASH risk. To explore the limitations of using NASHmap for NASH status prediction, we considered the potential causes underlying model errors and differences in performance between populations.

Misclassified patients present more subtle clinical changes than correctly classified patients. False negatives have profiles closer to non-NASH patients for several key features, including those suggesting normal liver health (AST, ALT, and albumin closer to or within normal range), normal metabolic function (HbA1C in normal range), or less inflammation (normal total protein). In false positives, these key features were similar to those of NASH patients (Table 4 and Supplemental Fig. 1). Interestingly, several measures of metabolic dysfunction did not appear to influence misclassification, and rates of hypertension were actually lowest in the false positive group.

There are several possible explanations for these misclassifications. NASHmap includes markers such as AST and ALT that define liver injury and inflammation. Normal AST and ALT levels have been noted for both NASH and NAFLD patients^11–13^, and a systematic review and meta-analysis consisting of 4084 patients reported normal ALT values in 25% and 19% of NAFLD and NASH patients, respectively^14^. NASH patients with less severe disease may simply have more marginal laboratory test values and predictions in these cases may have lower accuracy. For example, severity of NAFLD/NASH is correlated with levels of AST and ALT^12,15^, and lower levels of fibrosis in NAFLD patients as judged by FIB-4^16^ and elastography^17^ are correlated with lower HbA1c levels. Until more sensitive parameters or laboratory tests to detect NASH are available, patients with marginal clinical profiles will likely remain more difficult to identify.

NASHmap performance can vary between populations. Differences in consistency of clinical data collection methods, frequency of data collection, diagnosis accuracy and population heterogeneity between data sources will impact performance. Consistent with this, a T2DM subcohort of NIDDK NASH patients had a higher percentage of correct classification (86%) than the full cohort (81%). Notably, 77% of non-T2DM patients were correctly predicted by NASHmap. This demonstrates good performance of the model even in subpopulations without diabetes, despite HbA1c being the model feature with the highest predictive importance^8^.

NASHmap showed its best performance when patient data for all 14 features were available. An initial assessment for missing HbA1c data showed that NASHmap performance decreases slightly after imputation of missing values. To reduce the impact of missing data, a patient-level imputation such as kNN was preferable. If all values are missing, imputation with a reasonable fixed value such as a median or mean in a cohort with similar patient characteristics is possible but shows a larger impact on performance.

NASHmap identified a large number of predicted NASH patients (31%; 883,867 out of 2,886,653) in a real-world database cohort with NAFLD-associated conditions but no recorded diagnosis of NASH or NAFLD. A similar percentage of NASH patients (35%) was recently predicted by a model in an at-risk population with T2DM and NAFLD^18^. In our study cohort, many of the predicted NASH patients are likely to be NASH but not yet diagnosed despite their above normal laboratory values and risk profiles, illustrating the underdiagnosis of NAFLD and NASH in the real world. Although the predicted number will inevitably include false positives, setting a high prediction cutoff to reduce false positives and minimize unnecessary testing will result in an increase in false negatives and many overlooked NASH patients. The prediction cutoff can be changed to balance these two groups as needed, depending on the NASH risk in the population being tested and the goal for predictions. Since false negatives displayed fewer features with clinical values outside of normal range, identifying them could be challenging regardless of the method used.

The strength of the current study is the use of the large OPTUM EHR dataset to determine the performance of NASHmap in a broad range of patients and explore NASHmap’s utility beyond the well-characterized NIDDK patient dataset used for model training. Limitations include the inability to make a definite diagnosis of NASH in the OPTUM dataset due to the unavailability of liver biopsy information and the large time frame during which cases were collected, since differences in NAFLD/NASH diagnosis rates and modalities may have occurred over time.

**Figure 1:**
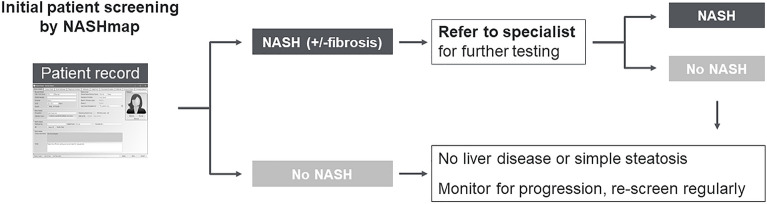
Initial NASHmap screening of patients for referral decision-making.

A possible application of NASHmap is integration as a tool in electronic health record databases to automate screening for patients at risk of NASH (Fig. 1). In summary, NASHmap has good performance using regular clinical and laboratory parameters available in electronic records and could be utilized in clinical practice to complement clinical assessment, improving referral of patients at high risk of NASH to specialists for care.

## Supplementary Information

Supplementary Information.

## Data Availability

Data from the NIDDK NAFLD Adult Database used here are available for request at the NIDDK Central Repository (NIDDK-CR) website, Resources for Research (R4R), https://repository.niddk.nih.gov/. Optum EHR data is available subject to payment of data fees to Optum.
