# Supplementary material for: NASHmap: clinical utility of a machine learning model to identify patients at risk of NASH in real-world settings
Source: Sci Rep. 2023 Apr 5;13:5573. doi: 10.1038/s41598-023-32551-2 (PMC10076319; doi:10.1038/s41598-023-32551-2)
Supplement: Supplementary file 1 — Supplementary Information. [file 41598_2023_32551_MOESM1_ESM.docx]

**Supplementary material**

**NASHmap: clinical utility of a machine learning model to identify patients at risk of NASH in real-world settings**

Jörn M. Schattenberg^1^, Maria-Magdalena Balp^2^, Brenda Reinhart^3^, Andreas Tietz^2^, Stephane A. Regnier^4^, Gorana Capkun^2^, Qin Ye^5^, Jürgen Loeffler^2^, Marcos C. Pedrosa^4^ and Matt Docherty^5^

^1^Metabolic Liver Research Program, Department of Medicine, University Medical Center, Mainz, Germany

^2^Novartis Pharma AG, Basel, Switzerland

^3^ZS Associates, Zurich, Switzerland

^4^Novartis Pharma AG, Basel, Switzerland (at the time of the study)

^5^ZS Associates, Philadelphia, PA, USA

**Corresponding Author Information**

Jörn M. Schattenberg

Professor

Department of Medicine

University Medical Center

Mainz, Germany

Email: [Joern.Schattenberg@unimedizin-mainz.de](mailto:Joern.Schattenberg@unimedizin-mainz.de)

**Supplementary Methods**

Subject Criteria for NIDDK NAFLD Training Dataset

*Inclusion*

1. Age ≥18 years at the time of first screening AND
2. NAFLD, NASH, or cryptogenic cirrhosis diagnosis
   1. Histological diagnosis of cryptogenic cirrhosis (histology must be confirmed by local NASH clinical research network (CRN) pathologist
   2. Suspected NAFLD based on imaging studies plus at least 1 of the following:

imaging evidence of portal hypertension (splenomegaly, portosystemic collaterals); albumin <3.5 g/dl; INR >1.3; platelet count <140,000 cell/μl; esophageal or gastric varices on endoscopy; ascites

*Exclusion*

1. Clinical or histological evidence of alcoholic liver disease
2. Excessive alcohol consumption during the 2 years prior to entry (males: 20 g/day, females: 10 g/day)
3. Evidence of other forms of chronic liver disease
4. Suspected or confirmed hepatocellular carcinoma (HCC)
5. Other conditions likely to interfere with the study

**Subject Criteria for Optum Dataset**

*Inclusion*

1. Age ≥18 years at the beginning of the patient's data window
2. Any of the following:
   1. Diagnoses (by International Classification of Disease (ICD) 9, 10 codes): NASH, NAFLD, cirrhosis, fibrosis of liver, HCC, unspecified fatty liver, polycystic ovarian syndrome, autoimmune hepatitis, obesity, type 2 diabetes, or hypertension
   2. Procedures (by procedure code): liver biopsy, liver transplant, or bariatric surgery
   3. Mention of these specific terms in physician notes or lab values indicative of these conditions
3. ≥6 months of medical history
4. ≥1 value for the clinical biomarkers used in NIDDK modeling at any point in the study period

*Exclusion*

1. Diagnosis of any of the following: alcohol dependence or history, chronic viral hepatitis C, alcohol withdrawal, alcohol-induced cirrhosis liver, hemochromatosis, cystic fibrosis, Wilson's disease, alpha-1-antitrypsin deficiency, or primary biliary cirrhosis
2. Positive tests for: hepatitis C antibody, hepatitis C, or hepatitis B
3. Certain medications including folic acid, thiamine, acamprosate, ledipasvir, ribavirin

**Supplementary Tables**

**Supplemental Table 1. Feature Values Outside of Normal Range**

| **Feature** | **Range or ULN** | **Outside of Normal Range** |
| --- | --- | --- |
| **Hemoglobin A1c (%) [Ref 1]** | <5.7 | ≥5.7 |
| **AST (U/L) [Ref 2]** | 0-35 | >35 |
| **ALT (U/L) [Ref 2]** | 0-35 | >35 |
| **Total protein (g/dL) [Ref 2]** | 6.0-7.8 | >7.8 |
| **AST/ALT [Ref 3]** | ≤1 | >1 |
| **BMI (kg/m^2^) [Ref 4]** | <30 | ≥30 |
| **TG (mg/dL) [Ref 2]** | 250 (desirable) | >250 |
| **Height (cm)** | NA | NA |
| **PLT (x10^3^/μL) [Ref 2]** | 150-350 | <150 |
| **WBC (x10^3^/μL) [Ref 2]** | 4-10 | >10 |
| **Hematocrit (%)[Ref 2]** | Male: 41-51  Female: 36-47 | <41  <36 |
| **Albumin (g/dL) [Ref 2]** | 3.5-5.5 | <3.5 |
| **Hypertension (%)** | NA | NA |
| **Female (%)** | NA | NA |

HbA1c=glycated hemoglobin; AST=aspartate transaminase, ALT=alanine transaminase; BMI=body mass index; TG=triglycerides; PLT=platelets; WBC=white blood cells

**Supplementary Figures**

**Supplemental Figure 1. AST, ALT, and HbA1c profiles in misclassified patients.** Box and whisker plots expressing median and interquartile range data for ALT, AST, HbA1c, and total protein.


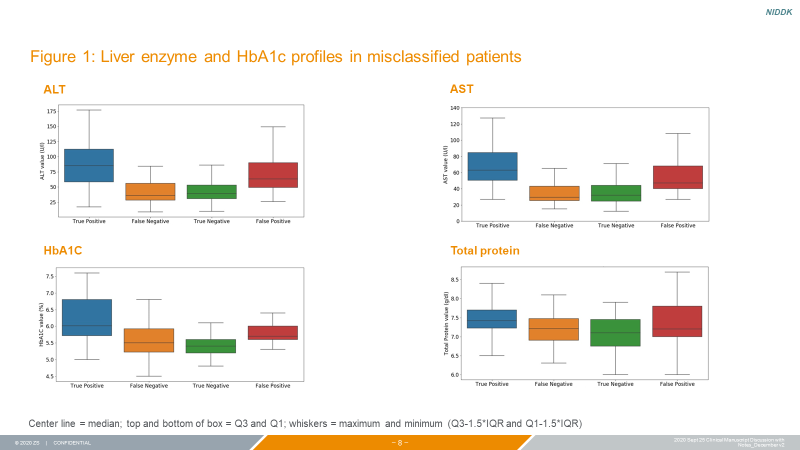


**Supplementary References**

1. American Diabetes Association; 2. Classification and Diagnosis of Diabetes: Standards of Medical Care in Diabetes-2020. *Diabetes Care* **43** (Supplement_1):S14-S31 (2020).

2. Reference Ranges. In: American College of Physicians. [Reference Ranges | ACP Internal Medicine Meeting (acponline.org)](https://annualmeeting.acponline.org/educational-program/reference-ranges) (2021).

3. Kasarala, G. & Tillmann, H.L.. Standard liver tests. *Clin. Liver Dis.* (Hoboken) **8**:13-18 (2016).

4. Garvey, W.T., Mechanick, J.I., Brett, E.M., Garber, A.J., Hurley, D.L., Jastreboff, A.M., Nadolsky, K. *et al*. American Association of Clinical Endocrinologists and American College of Endocrinology Comprehensive Clinical Practice Guidelines for Medical Care of Patients with Obesity. *Endoc.r Pract*. **22** Suppl 3:1-203 (2016).
